# Environmental Niche Overlap between Common and Dusky Dolphins in North Patagonia, Argentina

**DOI:** 10.1371/journal.pone.0126182

**Published:** 2015-06-19

**Authors:** Guillermo Martín Svendsen, María Alejandra Romero, Gabriela Noemí Williams, Domingo Antonio Gagliardini, Enrique Alberto Crespo, Silvana Laura Dans, Raúl Alberto González

**Affiliations:** 1 Instituto de Biología Marina y Pesquera Almirante Storni (IBMPAS) / Escuela Superior de Ciencias Marinas (ESCiMar), Universidad Nacional del Comahue, San Antonio Oeste, Río Negro, Argentina; 2 Consejo Nacional de Investigaciones Científicas y Técnicas (CONICET), Ciudad Autónoma de Buenos Aires, Buenos Aires, Argentina; 3 Laboratorio de Mamíferos Marinos, Centro Nacional Patagónico (CENPAT, CONICET), Puerto Madryn, Chubut, Argentina; 4 Unidad de Oceanografía y Meteorología, Centro Nacional Patagónico (CENPAT, CONICET), Puerto Madryn, Chubut, Argentina; 5 Instituto de Astronomía y Física del Espacio (IAFE, CONICET), Ciudad Autónoma de Buenos Aires, Buenos Aires, Argentina; CNRS, FRANCE

## Abstract

Research on the ecology of sympatric dolphins has increased worldwide in recent decades. However, many dolphin associations such as that between common dolphins (*Delphinus delphis*) and dusky dolphins (*Lagenorhynchus obscurus*) are poorly understood. The present study was conducted in the San Matías Gulf (SMG) ecosystem (North Patagonia, Argentina) where a high diet overlap among both species was found. The main objective of the present work was to explore the niche overlap of common and dusky dolphins in the habitat and temporal dimensions. The specific aims were (a) to evaluate the habitat use strategies of both species through a comparison of their group attributes (social composition, size and activity), and (b) to evaluate their habitat preferences and habitat overlap through Environmental Niche modeling considering two oceanographic seasons. To accomplish these aims, we used a historic database of opportunistic and systematic records collected from 1983 to 2011. Common and dusky dolphins exhibited similar patterns of group size (from less than 10 to more than 100 individuals), activity (both species use the area to feed, nurse, and copulate), and composition (adults, juveniles, and mothers with calves were observed for both species). Also, both species were observed travelling and feeding in mixed-species groups. Specific overlap indices were higher for common dolphins than for dusky dolphins, but all indices were low, suggesting that they are mainly segregated in the habitat dimension. In the case of common dolphins, the best habitats were located in the northwest of the gulf far from the coast. In the warm season they prefer areas with temperate sea surface and in the cold season they prefer areas with relatively high variability of sea surface temperature. Meanwhile, dusky dolphins prefer areas with steep slopes close to the coast in the southwestern sector of the gulf in both seasons.

## Introduction

Research on the ecology of sympatric dolphins has increased worldwide in recent decades [1–3]. This is in part because dolphins, together with primates, are the mammalian groups with the most highly elaborated brains and with most ecologically and socially complex populations [4, 5]. Therefore, the ways in which these populations share or compete for resources are also expected to be highly complex. However, detailed ecological studies on sympatric dolphins are currently scarce in comparison with primate studies. Moreover, many dolphin associations around the world have not yet been well studied [5].

In the lasts years, researchers focused on understand how species overlap (or segregate) in different niche dimensions [3, 5, 6]. Many of them agree that similar species of dolphins co-occurring in the same immediate habitat avoid competition through segregation, at least, in one of three main niche axes: habitat (influence of key environmental factors defining the spatial distribution), diet (diet composition, trophic level and prey quality) and time (use of habitat and resources according to time, such as seasons and time of day) [3, 5, 6]. So, different approaches have been recently explored and applied to dolphins and other sympatric species to measure niche overlap [2, 3, 6–8]. The estimation of overlap indices through the outputs of Environmental Niche Models (ENMs) allows to take advantage of historical and/or occasional data of species presence [6–8]. This is important considering the complexity and coast of data collection for dolphin ecology in marine environments (the main reason of information gaps about dolphin associations in many world regions) [1, 9, 10].

One association of dolphin species that has not yet been well studied is that between dusky dolphins (*Lagenorhynchus obscurus*) and common dolphins (*Delphinus delphis*). Both are amongst the highly encephalized dolphin species [11] and consequently we expect them to have complex patterns of sympatry. The co-occurrence of these species has been reported (and briefly described) in New Zealand [12–14], southern Peru [15] and northern Patagonia, Argentina [16, 17]. Ecology of dusky dolphins from Patagonia is well known [18]. The habitat of this species was recently modeled at a high spatial resolution in Golfo Nuevo and at a lower resolution over a large area of coastal and neritic waters of Patagonia [19–21]. On the other hand preliminary studies of the distribution patterns of common dolphins have been conducted in the SMG [22, 23], however, ecological aspects such as their habitat use, activity patterns and group characteristics are poorly known.

**Fig 1:**
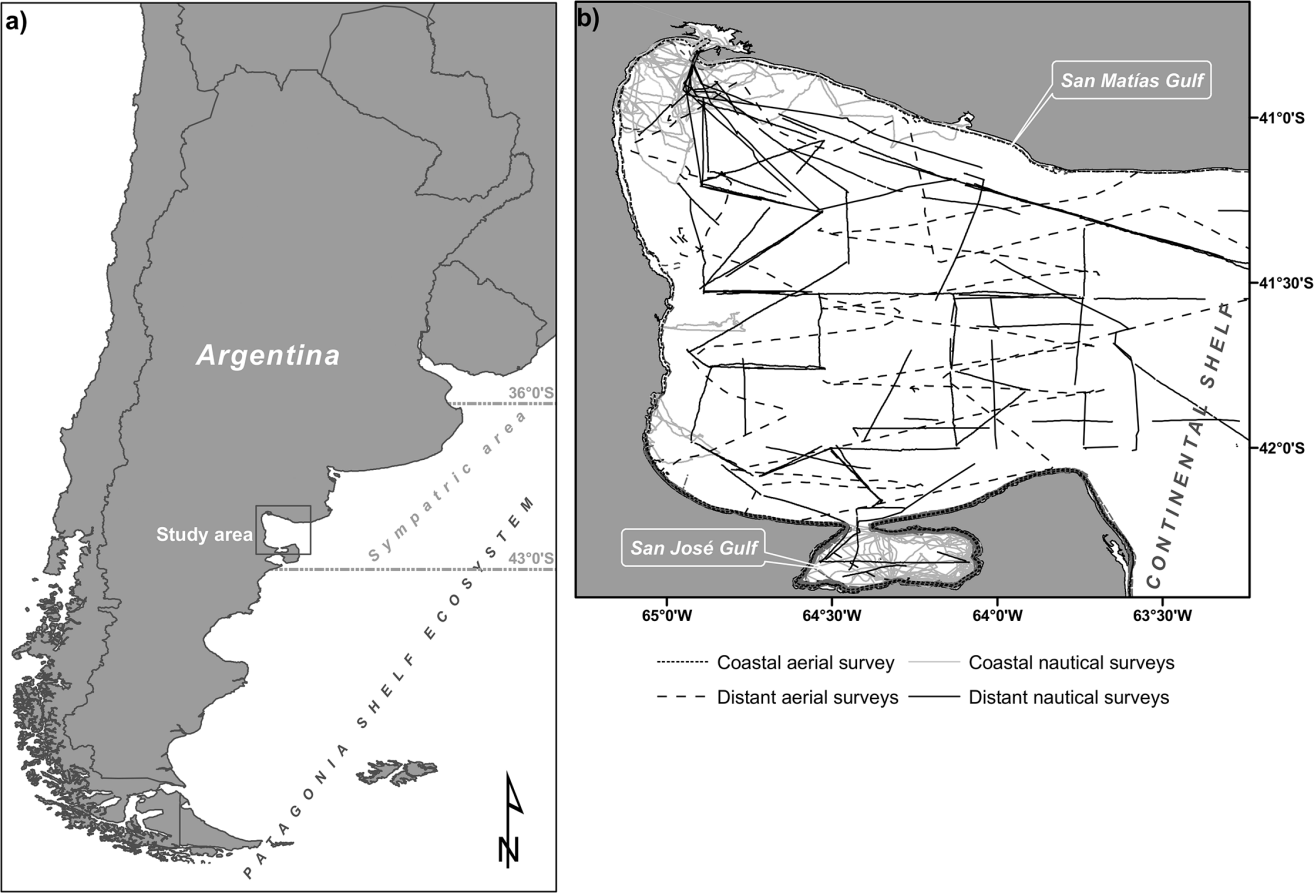
Study Area. (a) Map showing the location of the study area and latitudinal limits of the sympatric area of common dolphins (*Delphinus delphis*) and dusky dolphins (*Lagenorhynchus obscurus*) in the Patagonia Shelf Ecosystem. (b) Map showing systematic surveys (transects) conducted between 1995 and 2011 in the study area.

According to studies of the distribution of each species, the sympatric area of common and dusky dolphins in the Patagonia Shelf Ecosystem is between 36°S (northern limit of the distribution of dusky dolphins) and 43°S (southern limit of common dolphins; Fig 1) [12, 19, 24–28]. In this area, particularly in the San Matías Gulf (SMG, 40°50’S to 42°15’S and 63°05’W to 65°10’W; Fig 1), both species occur throughout the year, eventually co-occur in the same environmental patches (they form mixed-species groups) and have a high diet overlap-the main prey of both dolphin species is the Argentine anchovy (*Engraulis anchoita*) [16].

Also, both species are caught by fisheries, mostly by mid-water trawls. This fishing gear appears to have the highest dolphin bycatch rates in Patagonia [27, 29]. This kind of trawls when directed to southern anchovy causes the incidental mortality of both dolphin species, sometimes in the same tow. In recent years in the SMG has been growing interest in the development of a pelagic fishery (mid-water trawl), with emphasis on anchovy catch [29]. Consequently, the development of such a fishery in the SMG might have serious consequences for pelagic dolphins. Moreover, on the North Patagonia shelf (off SMG), there is an overlap in the size range of anchovies consumed by dolphins and the size range of anchovies targeted by the pelagic fishery [15]. Therefore, if this fishery expands into the SMG, there could potentially be direct competition with the dolphins [15].

In this context, the association of common and dusky dolphins in SMG is an interesting model to study patterns of dolphin sympatric ecology. Under the assumption of limited food resources, since common and dusky dolphins occur through all the year in the same geographic area and are highly overlapped in the diet dimension, we hypothesize that they are segregated in the habitat and/or time axes to avoid competition. Precisely, we propose that they might be using the same habitat in different ways (i.e. different habitat use strategies [1, 30]) or they might be segregating in different habitats within the SMG (i.e. they differ in their habitat preferences [30]).

Therefore the main objective of the present work was to explore the niche overlap of common and dusky dolphins in the habitat and time (in a seasonal scale) dimensions. For the SMG, we have a historical database of records of both species from different sources (many of which are occasional sightings), which allowed us to conduct the following specific objectives: a) to evaluate the habitat use strategies of both species through the comparison of their group attributes (social composition, size and activity), and b) to evaluate their habitat preferences and habitat overlap through Environmental Niche modeling in different seasons of the year.

## Methodology

### Study area

**Fig 2:**
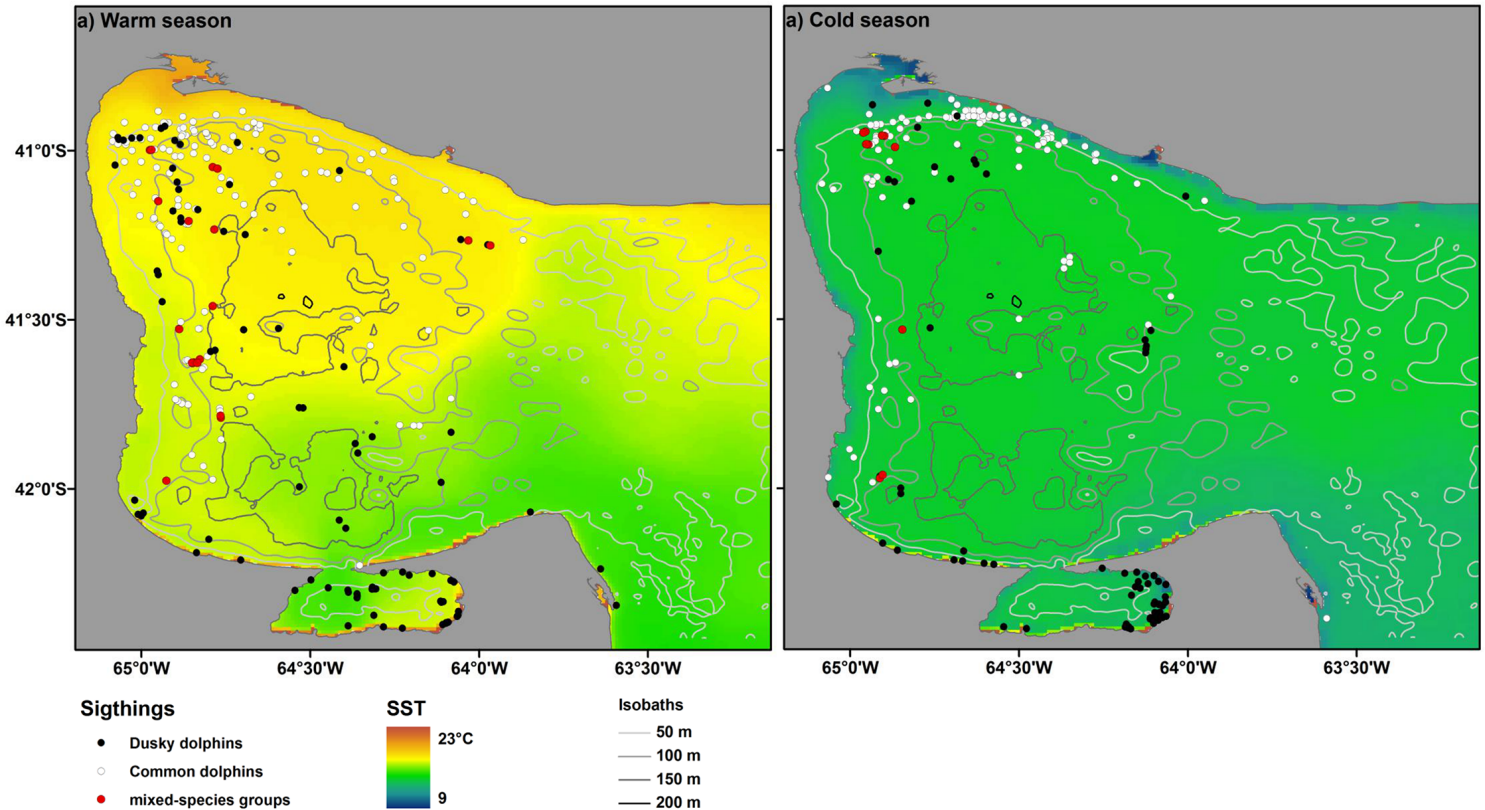
Sightings of common dolphins (*Delphinus delphis*), dusky dolphins (*Lagenorhynchus obscurus*) and mixed-species groups. (a) Warm season and (b) cold season. Study period (1983–2011). Maps also show means SST gradients obtained from AVHRR (NOAA) satellite images of each study season.

The study area is located in the North Patagonia Shelf Ecosystem (Argentina) and covers a total area of 23,700 km^2^, which includes the SMG, an area of the continental shelf next to the mouth of the SMG, and the San Jose Gulf (SJG) (Fig 1). In the present study, we refer to this combined area as the *SMG ecosystem*. The SMG is a semi-closed basin with a maximum depth of 200 m in the center of the basin. The continental shelf on the eastern side forms an open basin with depths ranging between 45 and 80 m [31, 32]. To the south, the SMG is connected to the SJG, which has an approximate area of 814 km^2^ and an average depth of 30 m [33]. The entire area forms a system with particular oceanographic and biological features, among which the formation of a thermal front from October to March is the most remarkable. This front divides the SMG into two water masses with different oceanographic conditions: the northwestern mass, which has a well-defined thermocline, high temperature (20.2°C, maximum monthly average) and salinity, low concentrations of nitrate and chlorophyll, and a low water exchange rate; and the southeastern mass, which lacks of vertical stratification, has lower values of temperature (18.6°C, maximum monthly average) and salinity, higher concentrations of nitrate and chlorophyll and is strongly influenced by the inflow of cold water from the south (Fig 2A) [34–37]. During the cold months (April to September), these properties tend to be more uniform throughout the basin (the mean temperature observed in both parts of the Gulf is close to 13°C; Fig 2B) [37].

Since this oceanographic process strongly influences the distribution of two important components of this ecosystem-primary producers and demersal fishery- [37, 38] we suspected that this might also affect directly or indirectly the distributions of pelagic dolphins. Therefore we conducted all comparative analyzes between both species of dolphins considering two seasons of the year-a) the warm season (October to March), when the thermal front forms in the SMG, and b) the cold season (April to September), when the thermal front is absent.

### Species data sets and group characterization

We collected data of common and dusky dolphins recorded during both systematic and occasional surveys (without measures of observational effort). Trained observers recorded occasional sightings from 1983 to 2010 during a) research surveys of demersal fish that covered the entire extent of the SMG onboard fishing vessels 30 to 40 m long, b) research surveys of bivalve mollusks and benthic fauna that covered the northwest coast of the SMG onboard research and fishing vessels 7 to 20 m long and c) regular surveys by the Fishery Observer Program (FOP) of the trawl demersal fishery of Río Negro Province (a survey area from 42° south latitude to the north of the gulf). This dataset includes reports of sightings as well as incidental catches of both dolphin species [16].

The systematic surveys (Fig 1) comprised different platforms of observation and sampling designs:

- Coastal nautical surveys, consisted of random transects between the coastline and a parallel line 20 km away and were conducted from inflatable boats (4 to 7 m long), traveling at an average speed of 9 kn with two or three observers on board. Study period 2006–2010.
- Distant nautical surveys, consisted of systematic transects onboard large vessels (30 to 70 m long) at an average speed of 10 kn and with one or two observers. These surveys covered areas further than 60 km from the coast. Study period: 2006–2011.
- Coastal aerial surveys, conducted with a high wing aircraft (Cessna B-182) and consisted of continuous transects parallel to the coast, approximately 500 m away from it, at an altitude of 152 m (500 feet) and an average speed of 90 kn. These surveys covered a coastal marine swath approximately 1000 m wide (500 m on each side of the plane trackline). Four people traveled on each flight: the pilot, one recorder and two observers (one on each side of the plane). Study period: 2004–2010.
- Distant aerial surveys, which consisted of zigzag transects previously defined to cover different depth ranges and water masses. These flights were performed with a high wing twin-engine aircraft (CASA 212) at an altitude of 152 m and a minimum speed of 110 kn. As in the distant nautical surveys, these surveys covered marine areas further than 60 km from the coast. As in the case of the coastal aerial surveys, two observers and one recorder traveled on each flight. Study periods: 1995, 2003, 2006–2008 and 2011.

The information gathered by observers during the occasional and systematic surveys included date, geographic position, social composition (except in aerial surveys) and number of individuals. If distances between individuals of the two dolphin species were less than 100 m, the group was classified as mixed-species group [39]. According to their social composition, groups were classified as adults and juveniles (A+J), mothers with calves (if more than 80% of the individuals were mothers with their calves, MwC) or a mixed combination of mothers with calves, juveniles and adults (A+J+MwC) [21]. Group size was assigned to one of the following categories: <10, 11–20, 21–50, 51–70, 71–100 or more than 100 animals. During the systematic nautical surveys, the predominant group activity at the time of the sighting was also recorded. The same is the activity in which most of the animals were engaged [40] and was classified into one of the following six categories: *feeding*, *traveling*, *socializing*, *resting*, *milling* [41], and *approach to the vessel*. During *feeding*, dolphins swam in circles and zigzags and enclosed a school of fishes. The presence of birds feeding together with or following the dolphins was a good indicator of feeding behavior. *Traveling* consisted of persistent and directional movement, where all group members swam synchronously. *Socializing* was characterized by interactions between individuals, usually in the form of body contact, with high-speed movements and frequent changes of direction and leaps. *Resting* consisted of a low level of activity during which the dolphins apparently floated motionless on the surface, with occasional slow forward movement. *Milling* consisted of low-speed movements with frequent changes in direction. *Approach to the vessel* consisted of dolphins traveling in the direction of the observational platform and, in many cases, interacting with it.

In order to evaluate habitat use strategies, we compared size, activity and social composition of groups between species for each seasons and between seasons for each species. We used Kolmogorov-Smirnov test to compare group size distributions and Chi-square tests for contingency tables to compare relative frequencies of activity and social composition. Each group attribute was analyzed individually because social composition and group activity were recorded only in the systematic nautical surveys, and during these surveys it was not always possible to assign all attributes to a single group.

### Environmental niche models

We used maximum entropy models as implemented in the Maxent 3.3.3k software (www.cs.princeton.edu/wschapire/maxent) to develop environmental niche models for both species. Maxent is a machine learning method that uses a set of occurrence localities (presence data) together with a set of environmental variables to produce a map representing the suitability of the environment for the species [42, 43]. This method has been used successfully to predict the distributions of common dolphins and other marine mammals [44, 45]. Maxent, as well as other models that only use presence records, is particularly useful to predict the geographic distribution of a species with low encounter rates across a large area [45–47].

The environmental variables included in the models were those for which information was available for the entire spatial extent of the study area (or for which it was feasible to make an accurate interpolation over the entire area) and that might contribute to the habitat prediction for the species. The variables selected were Depth, Seabed Slope, Distance to the Shoreline, Sea Surface Temperature (SST), and Standard Deviation of SST (SST-SD). These variables have been used as habitat predictors for dusky dolphins in Patagonia and for common dolphins in other regions of the world and at different spatial scales [19, 20, 45]. In certain cases, environmental variables are direct predictors of the distribution of the species because they can directly influence their physiological capabilities; in other cases, they may be indirect predictors because they influence the distribution of their prey or predators [10, 48].

To ensure that the selected environmental variables were not highly correlated, we calculated Pearson correlations before running the model. We tested correlation among variables directly using the raster files in the software package ENMTools Version 1.4.4 [49]. We considered that two variables were highly correlated if the coefficient of correlation between them was greater than 0.7 [50].

We obtained the SST data from AVHRR (NOAA) satellite images with a 1 × 1 km spatial resolution. The images used were provided by the Argentine National Commission of Space Activities (CONAE) for the period 2000–2008 and processed at the Remote Sensing Laboratory of the National Patagonian Center (CENPAT-CONICET, Unit of Oceanography and Meteorology). As whit the characterization of groups, we analyzed the distribution of each species separately during the warm and cold seasons. Accordingly, we constructed a mean SST map and an SST-SD map for each season. We constructed the depth map by interpolating bathymetric points from nautical charts (Naval Hydrographic Service, Argentina) and altimetric points of the coast (National Geographic Institute, Argentine) using an ordinary Kriging function. We obtained the slope map from the depth map by assigning to each pixel the maximum slope observed between itself and its eight neighboring pixels. We generated the distance map by calculating the minimum distance to the shoreline for each pixel. We used the resolution and spatial extent of the SST images for all raster layers. We performed all of these tasks in a cartographic information system previously designed for the study area [51].

We ran Maxent with auto features and using the cross-validation technique. To reduce the bias caused by the heterogeneous effort distribution over the study area, we set the software to remove duplicate presence records [52]. We set maxent to do 1000 iterations and to use 1000 background points in each run. The output format selected for model values was the logistic one, which can be interpreted as an index of habitat suitability as well as an estimate of the probability of species presence conditioned on environmental variables [53]. For each species and season we ran 25 model replications. Thus, we obtained a final mean and standard deviations models for each species and season. We evaluated the performance of each model with the AUC (area under the receiver operating characteristic curve) [54]. For each species and season we obtained mean and standard deviations of AUC values from the Maxent output.

We used the permutation importance index to identify the most important environmental variables for the species being modeled. This index is a measure of the contributions of each environmental variable to the fit of the final Maxent model and it is determined by randomly permuting the values of that variable among the training points (both presence and background) and by measuring the resulting decrease in the training AUC. A large decrease indicates that the model depends heavily on that variable. Values are normalized to obtain percentages (*A brief tutorial on MaxEnt* by Steven Phillips, AT&T Research http://www.cs.princeton.edu/~schapire/maxent/). We used the permutation importance values averaged over the 25 index values resulting from each model replication.

We evaluated habitat preference of the species in each season, through response curves of their most important environmental variables (i.e. the first two variables which together contributed in 50% or more to the fit of the model) and through maps of the four averaged models (one for each season of each species). Maxent performs each curve by generating a model using only the corresponding variable.

### Environmental niche overlap

We estimated the environmental niche overlap between common and dusky dolphins in the SMG ecosystem for each season from the mean Maxent models. Since the sightings of both species came from the same dataset, the survey coverage was equal for both species. We also assumed that they have the same detectability within the study area due to its similar size and its ability to form groups from few to hundreds of individuals. Therefore, it is probable that any detected difference in the modeled distributions of the species refers to real ecological differences between them [55].

We calculated specific overlap indices between species for each season. We used the specific overlap index SO_*ik*_ [56, 57], that calculates the probability of obtaining the utilization curve (of resources) of predator i from the utilization curve of predator k [56]. We chose this index because it has a statistical test associated. We constructed this index converting the suitability scores of Maxent to probability distributions on geographic space by dividing the individual cell values (*n*_*j*_) by the sum of all values (*N*) [7].The general formula of the index is:

$$SO_{ik}\;=e^{\left(\sum_{j=1}^{r}\left(p_{ij}\;\text{ln}\;p_{kj}\right)-\sum_{j=1}^{r}\left(p_{ij}\;\text{ln}\;p_{ij}\right)\right)}$$

where p_*ij*_ (or p_kj_) denotes the probability assigned by the ENM for species i (or k) to cell j, and r is the total number of cells with suitability scores. The null hypothesis that the specific overlap of species i onto k is complete (SO_*ik*_ = 1) was tested with the U-statistic [56]:

$$U_{ik}=-2N_{i}\;\text{ln}\;SO_{ik}$$

which follows a Chi-square distribution with (r-1) degrees of freedom.

## Results

### Group size, composition and activity pattern

The spatial distribution of effort of systematic surveys was heterogeneous but covered all extend of the study area (Fig 1). We compiled 302 sightings of common dolphin groups and 214 of dusky dolphin groups for the SMG ecosystem during the 1983–2011 period (Fig 2; Table 1). Twenty-five of those groups corresponded to mixed-species groups (17 observed during the warm season and 8 during the cold season; Fig 2). Most mixed-species groups (92%) were recorded during systematic nautical surveys (mixed-species groups represented 12% of the total groups sighted in those surveys). Statistical comparisons of group characteristics between seasons were not conducted for mixed-species groups due to the low sample size for each category.

**Table 1:** Total sightings obtained from opportunistic and systematic datasets and number of different localities (presence records without repetitions) used to run the models.

Species	Season	N° sightings		N° localities used to run models
		Opportunistic	Systematic	
*Delphinus delphis*	Cold	80	46	110
	Warm	38	138	148
*Lagenorhynchus obscurus*	Cold	12	85	72
	Warm	9	108	93

**Fig 3:**
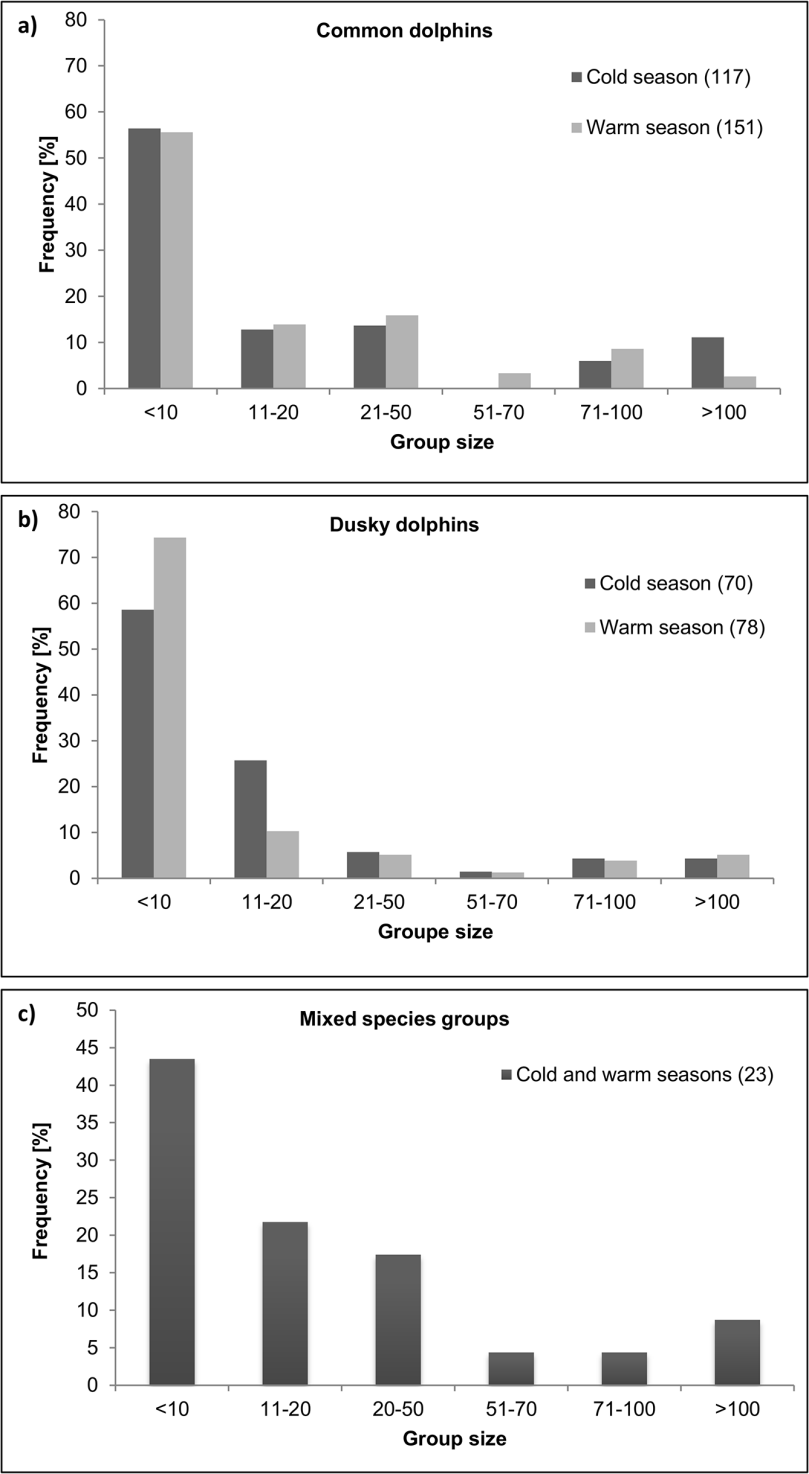
Group size distributions. Frequency distributions (percentages) of group size categories for (a) common dolphins (*Delphinus delphis*), (b) dusky dolphins (*Lagenorhynchus obscurus*) and (c) mixed-species groups of common and dusky dolphins. The number in parentheses indicates the sample size.

Each dolphin species exhibited the same group size distribution between the warm and cold seasons (Kolmogorov-Smirnov test, *P* > 0.05, in the two pairwise comparisons; Fig 3A and 3B), ranging from fewer than 10 individuals to more than 100 individuals. The group size distribution differed significantly between species only in the warm season (Kolmogorov-Smirnov test, *P* < 0.05). Groups with 10 or fewer individuals represented approximately 60% of the total number of common dolphin groups in both seasons, whereas this category represented almost 80% of all dusky dolphin groups in the warm season (Fig 3A and 3B). Mixed-species groups ranged from fewer than 10 to more than 100 individuals, and groups with 10 or fewer individuals were the most frequently observed group size (Fig 3C). In most mixed-species groups (n = 19), common dolphins were more abundant than dusky dolphins. In three groups, the two species were equal in number, and in other three groups, dusky dolphins outnumbered common dolphins. On average, mixed-species groups were composed of 68% (SE = 4.4%) common dolphins and 32% (SE = 4.4%) dusky dolphins. The largest mixed-species group observed was composed of 500–700 common dolphins and 50–70 dusky dolphins traveling together.

**Fig 4:**
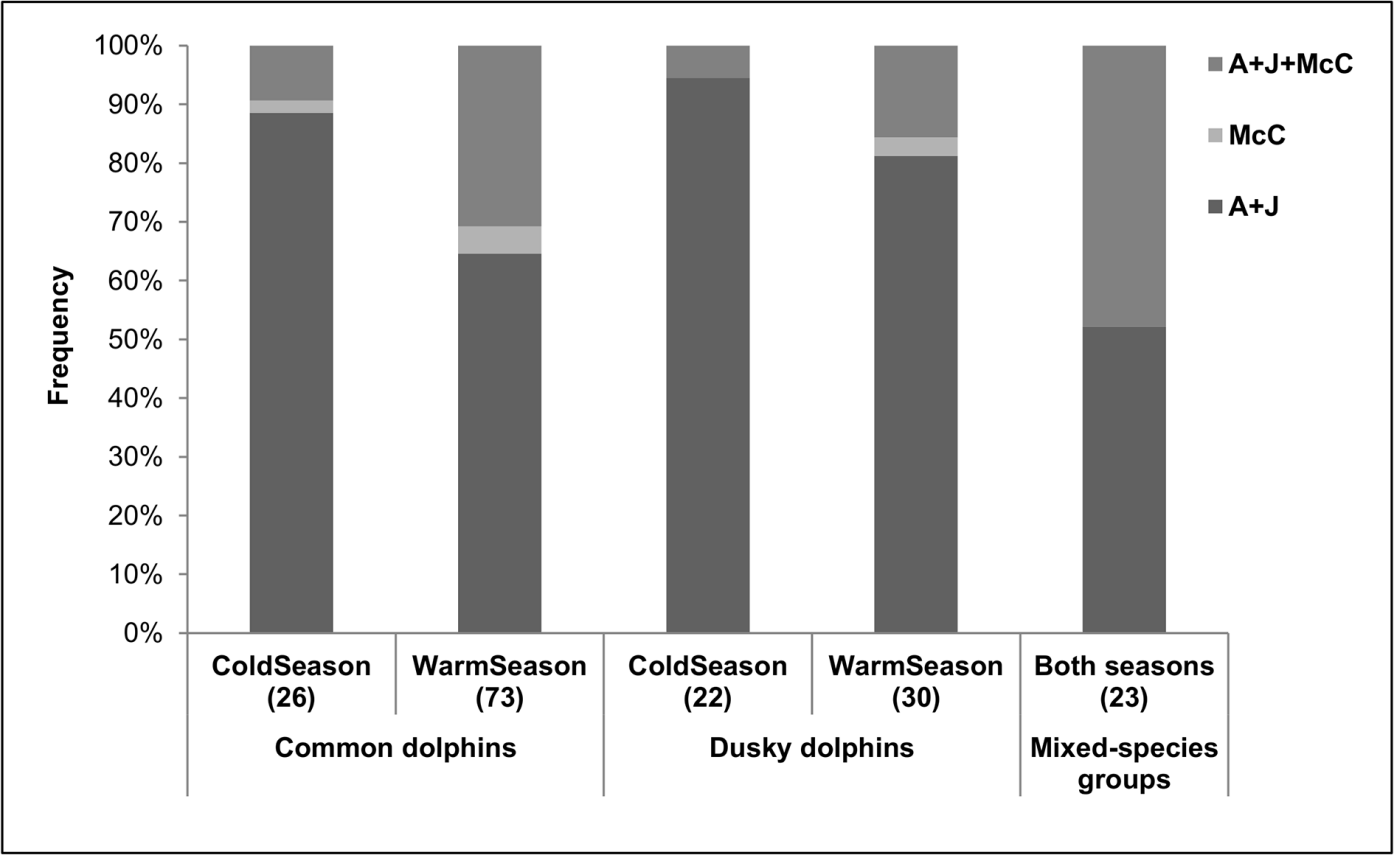
Social composition of groups. Percentages of occurrence of social categories for common dolphins (*Delphinus delphis*), dusky dolphins (*Lagenorhynchus obscurus*) and mixed-species groups recorded in each season. (A+J) adults and juveniles, (MwC) mothers with calves, (A+J+MwC) mixed combination of mothers with calves, juveniles and adults. The number in parentheses indicates the sample size.

Social composition of groups was similar between species for each season (Chi-square test, *X* ^*2*^ = 1.2, df = 2, *P* > 0.01 for the cold season; *X* ^*2*^ = 3.9, df = 2, *P* > 0.05 for the warm season; Fig 4). Groups including mothers with calves (MwC and A+J+MwC) were more frequent in the warm season than in cold season for both dolphin species (Fig 4). However, significant differences in social composition between seasons were found only for common dolphins (Chi-square test, *X* ^*2*^ = 1.9, df = 2, *P* > 0.05 for dusky dolphins; *X* ^*2*^ = 11.1, df = 2, *P* < 0.01 for common dolphins). All mixed-species groups were composed of adults and juveniles of both species (Fig 4). Seven of these groups had also common dolphin mothers with calves, and two groups had dusky dolphin mothers with calves. Mothers with calves of both species were never observed in the same mixed-species group.

**Fig 5:**
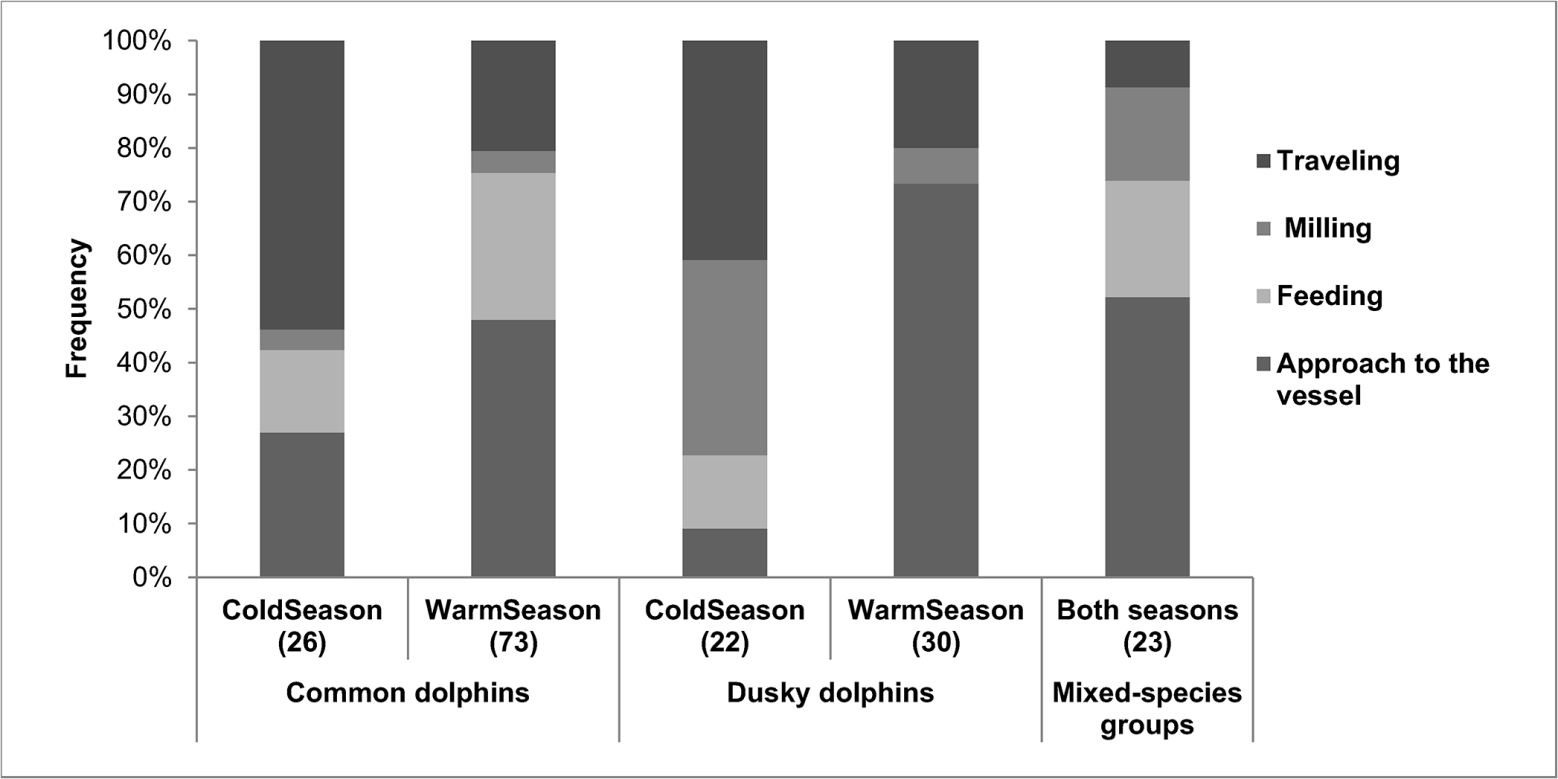
Predominant group activities. Percentages of occurrence of predominant group activities for common dolphins (*Delphinus delphis*), dusky dolphins (*Lagenorhynchus* obscurus) and mixed-species groups. Data were only obtained from systematic nautical surveys. The number in parentheses indicates the sample size.

*Traveling*, *milling*, *feeding* and *approach to the vessel* were the predominant group activities observed for both dolphin species in both seasons. *Resting* and *socializing* were never observed at the beginning of the sightings; however, *socializing* was sometimes observed opportunistically when the research boat spent more time close to the groups. This activity was recorded in two groups of dusky dolphins and in three groups of common dolphins and copulation events were observed in some of these cases. The relative observed frequency of each activity differed between seasons for each species and also between species for each season (Chi-square test, *X* ^*2*^ = 23.2, df = 3, *P* < 0.05 for dusky dolphins; *X* ^*2*^ = 10.4, df = 3, *P* < 0.05 for common dolphins; *X* ^*2*^ = 9.2, df = 3, *P* < 0.05 for cold season; *X* ^*2*^ = 11.0, df = 3, *P* < 0.05 for warm season; Fig 5). *Traveling* and *approach to the vessel* were the most frequent activities for both species during cold and warm seasons, respectively (Fig 5). *Feeding* was observed in all cases except in groups composed exclusively of dusky dolphins in the warm season. However, dusky dolphins were observed feeding with common dolphins in two sightings during that season. Most mixed-species groups were observed approaching to the research vessel (Fig 5). On some of those occasions, individuals of both species were observed swimming very near each other (< one body length of distance). These events lasted for a few to several seconds, and agonistic interactions were never observed. Feeding was the second most important activity observed in mixed-species groups, followed by milling and travelling (Fig 5).

### Environmental niche models

All the environmental variables were included in the models since none of them were highly correlated (Table 2). We obtained higher AUC values than expected by chance for the four resulting averaged models, (AUC > 0.5 in all cases) and the standard deviations were low, indicating uniformity amongst replications (Table 3).

**Table 2:** Spearman’s correlation coefficients among environmental variables.

		1	2	3	4	5	6	7
1	Depth	1						
2	Seabed Slope	0.09	1					
3	Distance to the Coast oastshoreline	0.46	-0.25	1				
4	SST cold season	0.69	0.03	0.38	1			
5	SST-SD cold season	-0.52	-0.15	0.05	-0.67	1		
6	SST warm season	0.12	-0.02	-0.14	-	-	1	
7	SST-SD warm season	0.16	-0.20	0.55	-	-	0.13	1

**Table 3:** AUC and variable importance values for each averaged model.

Species	Season	AUC		Permutation Importance				
		mean	Standarddeviation	Distance	Slope	Depth	SST-SD	SST
*Delphinus delphis*	Cold	0.868	0.079	**15.7**	6.5	14.3	**52.1**	11.3
	Warm	0.887	0.060	**16.4**	8.5	1.8	9.1	**64.2**
*Lagenorhynchus obscurus*	Cold	0.823	0.112	**43.2**	**43.8**	3.3	5.8	3.9
	Warm	0.778	0.104	**36.2**	10.5	**21.6**	12.4	19.3

The two most important variables are presented in bold.

**Fig 6:**
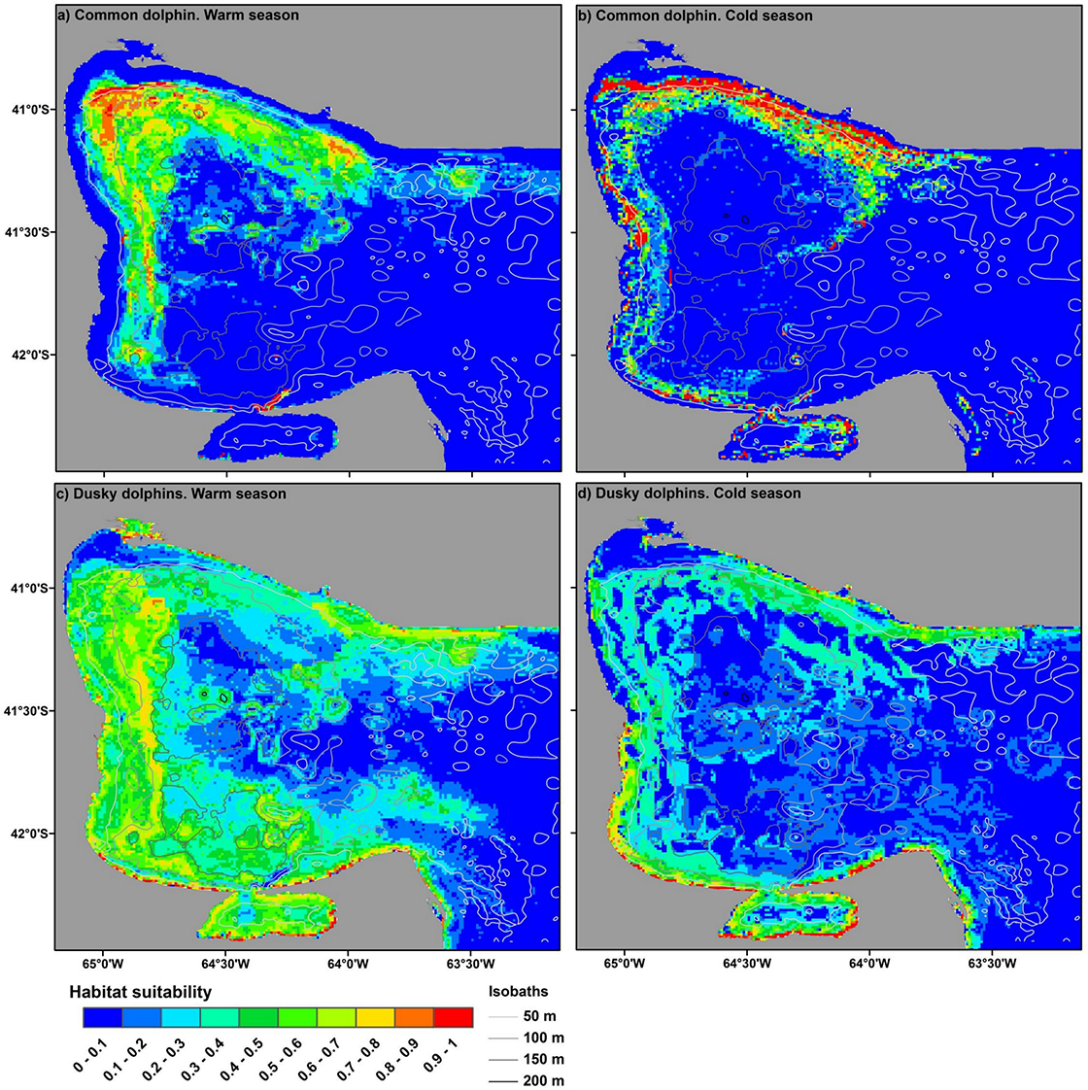
Averaged Maxent Models. Maps of mean habitat suitability resulting from MaxEnt modeling of common dolphins (*Delphinus delphis*, “a” and “b”) and dusky dolphins (*Lagenorhynchus obscurus* “c” and “d”) within the study area for each study season.

Resulting models predicted a more restricted distribution of habitats of medium to high suitability (> 0.3) for common dolphins than for dusky dolphins in both seasons considered (Fig 6). The most suitable habitats (> 0.7) for dusky dolphins were predominantly located along the southern coast of the study area, whereas those for common dolphins were predominantly located in the northwestern sector and away from the coast (Fig 6).

**Fig 7:**
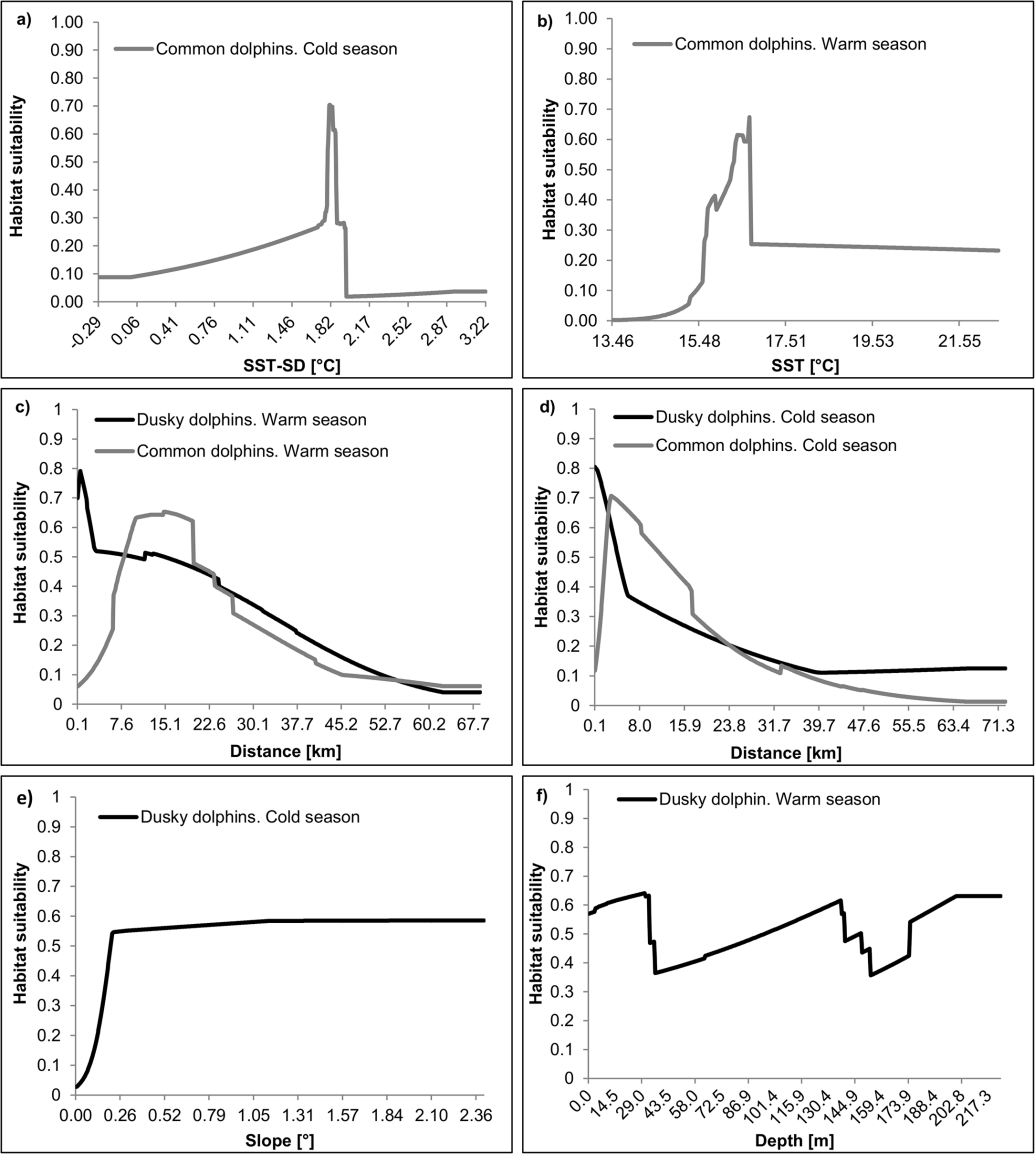
Response curves of dolphin habitat suitability to environmental variables. Averaged response curves of the two most important environmental variables resulting from Maxent modeling of common dolphins (*Delphinus delphis*) and dusky dolphins (*Lagenorhynchus obscurus*) localities. (a) Response curve of common dolphin habitat suitability to Standard Deviation of Sea Surface Temperature (SST-SD) in the cold season; (b) response curve of dusky dolphin habitat suitability to Seabed Slope in the cold season; (c and d) response curves of common and dusky dolphin habitat suitability to distance to the coast in the warm and cold season respectively (curves of both species are plotted together in order to facilitate visual comparisons); (e) response curve of common dolphin habitat suitability to Sea Surface Temperature (SST) in the warm season; (f) response curve of dusky dolphin habitat suitability to Depth in the warm season.

All environmental variables contributed to the fit of each model; however, variable contributions were different for each species and season modeled (Table 3). In the cold season the best habitats for common dolphins were located in waters with relatively high levels of SST-SD and relatively close to the coast (Fig 7A and 7C); while the best habitats for dusky dolphins were located in waters very close to the coast but with seafloor slopes from at least 0.21° (i.e. a drop of approximately 4 meters in a run of 1000m) to the maximum slope observed in the study area (i.e. a drop of approximately 37 m in a run of 1000 m; Fig 7B and 7C). In the warm season the best habitats for common dolphins were located in temperate waters (habitat suitability peaks at 18°C) and further from the coast than in the cold season (Fig 7C and 7D), while the best habitats for dusky dolphins were located very close to the coast as in the cold season, but with a staggered pattern of habitat suitability in response to depth (Fig 7D–7F).

### Environmental niche overlap

The SO_*ik*_ index indicated significant differences among both species (Table 4). The specific overlaps were higher for common dolphins than for dusky dolphins in both seasons. Each species presented their environmental niche more overlapped with the other in the warm season than in the cold season. We did not reject the null hypothesis that the specific overlaps of common dolphin on dusky dolphins are complete, but we reject the null hypothesis that the specific overlaps of dusky dolphins on common dolphins are complete in both seasons (Table 4).

**Table 4:** Environmental niche overlaps between common dolphin *Delphinus delphis* and dusky dolphin *Lagenorhynchus obscurus*, in both study seasons.

i	k	Season	SO_ik_	U	P
Common dolphin	Dusky dolphin	Cold	0.242	7360	= 1
		Warm	0.457	5317	= 1
Dusky dolphin	Common dolphin	Cold	0.029	27671	<0.001
		Warm	0.070	37973	<0.001

SO_ik_, specific overlap of species i onto species k; U, statistic to test the null hypothesis that the specific overlap of species i onto k is complete; P, probability of the statistic. The degrees of freedom are the same for the four test (= 25672).

## Discussion

In the SMG ecosystem, common dolphins and dusky dolphins conduct the principal activities of their life cycles: a) the presence of herds with calves indicates that both species use the area to nurse; b) *socializing*, including copulations, were sometimes observed in both species, which suggests they use the area to mate; and c) *feeding* was observed many times in both species, suggesting the importance of this activity within the study area. Moreover, we observed both species feeding, milling and travelling together. The occurrence of mixed-species groups (12% taking into account coastal nautical surveys only) is mid-level compared to the occurrence of mixed-species groups of different dolphin species in other regions (1.5% to 33.3% [58, 59]). This comparison suggests that mixed-species groups occur quite often in the SMG ecosystem. In general terms, all these observations suggest that both species may have similar habitat use strategies in the study area; however we found some significant differences among them.

The environmental niche overlap was higher for common dolphins than for dusky dolphins, and they were more overlapped in the warm season than in the cold one. Interestingly, this pattern is similar to that found for specific diet overlap in the SMG. Romero [60] found that common dolphins diet is more overlapped to dusky dolphins diet (SO = 0.91), than the overlap of dusky dolphins diet to common dolphins one (SO = 0.38). These high overlaps were because both dolphin species share their main prey, the Argentine anchovy, (for both species this prey has a relative importance higher than 82% of their diets)[16]. Therefore, environmental overlaps might be partially replicating the environments where schools of anchovies are available for both species.

On the other hand, specific habitat overlap was much lower than diet overlap. Estimated distributions showed that the best habitats for each species are spatially separated (Fig 6). These results support the hypothesis that if food resources are limited, they might be mainly segregating in the habitat dimension. Habitat partitioning has been suggested as a strategy to avoid competition and thus promote coexistence in sympatric dolphins that show a significant diet overlap [1, 2]. According to different cases of sympatric dolphins, habitat segregation may occur at different spatial and temporal scales [5]. In our study, segregation occurs mostly on a regional scale and it changes seasonally. This pattern suggests that dolphins might be changing their feeding strategies according to seasonal changes of their prey. Although we do not have data of the seasonal patterns of abundance and distributions of pelagic fish to include in our models, we can infer some feeding strategies performed by each dolphin species from the results obtained here and in other studies.

The modeled distributions of dusky dolphins in the SMG might be resulting for a combining effect of feeding and predators avoidance strategies as was suggested in previous studies in Nuevo Gulf and the San José Gulf [20, 61, 62]. The most important environmental variables affecting the modeled distributions of dusky dolphins were distance to the coast, depth and seabed slope (Table 3). The response curves of these variables suggest that dusky dolphins preferred areas with steep slopes close to the coast. A combining effect of both factors has been found and well described for dusky dolphins in Kaikoura, New Zealand. In this region, Dusky dolphins move offshore during late afternoon and evening to feed on the rising scattering layer, and return to the coast to rest and socialize during the day [63]. If dusky dolphins follow the same pattern in the SMG, this may be a reason why we did not observe many feeding activities during the warm season. Added to these observations, in the warm season dusky dolphins exhibited a higher proportion of groups composed of less than 10 individuals than common dolphins. This finding may indicate a broader dispersion of dusky dolphins groups in response to a potential dispersion of the food in that season.

Meanwhile, the feeding strategy of common dolphins might be closely associated with the occurrence of high biological productivity areas resulting from upwelling events. The distribution of common dolphins in the cold season is strongly influenced by SST-SD (Table 3). Habitat suitability for this species peaked at relatively high levels of that variable (Fig 7A). Recent investigations showed that northerly and westerly winds produce upwelling events of few days over the west and north coasts of the SMG respectively [64–66]. These events can be observed in daily SST satellite images as zones of lower temperature than their surrounding waters and occur around 27 times a year [66]. Therefore, over a period of few months (such as the seasons considered here), areas of upwelling should be spatially correlated with zones of high SST variability (in our study SST-SD is an indicator of this seasonal variability). Notably, upwelling events over the west coast occurred more frequently in the cold season (about 17 events per year) than in the warm season (about 10 events per year) [66]. So, this oceanographic process might be conditioning the distribution of common dolphin preys in the cold season.

Additionally, the co-occurrence of common and dusky dolphins in the study area would be favored by the confluence of two different water masses in the SMG ecosystem. Each of these water masses has physicochemical properties that have been described as preferred by each of these dolphin species. Common dolphins are distributed mostly in warm temperate and salty waters of tropical and mid-latitudes of both hemispheres, whereas dusky dolphins are usually distributed in cold to temperate and generally less saline waters of the southern hemisphere [12–14, 67, 68]. Thus, the cold and less saline water entering the SMG ecosystem from the Patagonian Coastal Current (sub-Antarctic origin) [69, 70] might favor the presence of dusky dolphins in the study area, whereas the warmer and more saline water mass that originates in the northwest of the SMG ecosystem [36, 37] would favor the presence of common dolphins. This pattern has also been suggested for New Zealand, where common dolphins reach their southern distribution limit (44°S) due to the warm subtropical water of the East Cape Current, and the occurrence of dusky dolphins is associated with a cold tongue of water from the Canterbury Current [14]. In the SMG ecosystem, the hypothesis is supported also by the following observations and results. First, large groups of common dolphins appear to reach the limit of their distribution in this gulf. Groups of common dolphins are sporadically observed in Golfo Nuevo (43°S) and never comprise more than 12 individuals (Dra. Mariana Degrati, personal communication; Laboratorio de Mamíferos Marinos. Centro Nacional Patagónico (CONICET). Boulevard Brown 2915, Puerto Madryn, Chubut, Argentina). The most suitable habitats for common dolphins estimated in this study were located primarily in the northwestern sector of the SMG ecosystem-corresponding to the warm and saline water mass region (Figs 2A and 6). In the warm season, SST was the most important variable to predict the distribution of common dolphins (Table 3). Therefore, we may infer that common dolphins prefer temperate waters of the SMG ecosystem (at least in the warm season).

## Conclusion

From previous studies we knew that common dolphins and dusky dolphins presented a considerable but asymmetrical diet overlap in the SMG. From this study we know now that these species also exhibit the same pattern of environmental overlap, i.e. in both cases specific overlap indices are higher for common dolphins than for dusky dolphins. However, environmental overlap indices are lower than diet ones. Therefore we conclude that in the SMG common and dusky dolphins are mainly segregated in the habitat dimension of their niches. Segregation occurs mostly on a regional scale and it changes seasonally. The best habitats for common dolphins are located in the northwestern sector of the gulf in areas separated from the coast. In the warm season common dolphins prefer areas with temperate sea surface and in the cold season they prefer areas with relatively high variability of SST. Meanwhile, dusky dolphins prefer areas with steep slopes that are close to the coast in the southwestern sector of the gulf both in warm and cold seasons.

We have presented the first study of the sympatric ecology of common and dusky dolphins. We have also described for the first time the size, social composition and activity patterns of mixed-species groups of these species and single-species groups of common dolphins in the Patagonian shelf ecosystem. Our results provide a platform for future research on the mechanism underlying dolphin distribution in north Patagonian gulfs. Our future studies will be focused on the distribution and abundance of dolphin prey, and on dolphin foraging behaviors on a finer spatiotemporal scale.
